# Physiotherapy Rehabilitation Post Patellar Dislocation (PRePPeD)—protocol for an external pilot randomised controlled trial and qualitative study comparing supervised versus self-managed rehabilitation for people after acute patellar dislocation

**DOI:** 10.1186/s40814-023-01349-4

**Published:** 2023-07-10

**Authors:** Colin Forde, Matthew L. Costa, Jonathan A. Cook, Elizabeth Tutton, Duncan Appelbe, Marloes Franssen, Rupert Barker, David J. Keene

**Affiliations:** 1 Nuffield Department of Orthopaedics, Rheumatology and Musculoskeletal Sciences, University of Oxford, Oxford, UK; 2 Patient and Public representative, Oxford, UK; 3 Exeter Medical School, University of Exeter, Exeter, UK

**Keywords:** Kneecap, Patellar instability, Patellofemoral joint dislocation, Patellar dislocation, Paediatric, Young people, Physical therapy, Physiotherapy, Non-surgical, Non-operative

## Abstract

**Background:**

Patellar dislocations mainly affect adolescents and young adults. After this injury, patients are usually referred to physiotherapy for exercise-based rehabilitation. Currently, limited high-quality evidence exists to guide rehabilitation practice and treatment outcomes vary. A full-scale trial comparing different rehabilitation approaches would provide high-quality evidence to inform rehabilitation practice. Whether this full-scale trial is feasible is uncertain: the only previous trial that compared exercise-based programmes in this patient population had high loss to follow-up. This study aims to assess the feasibility of conducting a future full-scale trial comparing the clinical and cost-effectiveness of two different rehabilitation approaches for people with an acute patellar dislocation.

**Methods:**

Two-arm parallel external pilot randomised controlled trial and qualitative study. We aim to recruit at least 50 participants aged ≥ 14 years with an acute first-time or recurrent patellar dislocation from at least three English National Health Service hospitals. Participants will be randomised 1:1 to supervised rehabilitation (four to six, one-to-one, physiotherapy sessions of advice and prescription of tailored progressive home exercise over a maximum of 6 months) or self-managed rehabilitation (one physiotherapy session of self-management advice, exercise, and provision of self-management materials). Pilot objectives are (1) willingness to be randomised, (2) recruitment rate, (3) retention, (4) intervention adherence, and (5) intervention and follow-up method acceptability to participants assessed through one-to-one semi-structured interviews (maximum 20 participants). Follow-up data will be collected 3, 6, and 9 months after randomisation. Quantitative pilot and clinical outcomes will be numerically summarised, with 95% confidence intervals generated for the pilot outcomes using Wilson’s and exact Poisson methods as appropriate.

**Discussion:**

This study will assess the feasibility of conducting a full-scale trial comparing supervised versus self-managed rehabilitation for people after acute first-time or recurrent patellar dislocation. This full-scale trial’s results would provide high-quality evidence to guide rehabilitation provision for patients with this injury.

**Trial registration:**

ISRCTN registry ISRCTN14235231. Registered on 09 August 2022.

**Supplementary Information:**

The online version contains supplementary material available at 10.1186/s40814-023-01349-4.

## Background

Patellar dislocations normally occur in a lateral direction. Most are non-contact injuries [1] and 66% occur during sport [2]. The reported incidence of first-time patellar dislocations is 2.6 to 42 per 100,000 person years at risk, highest in adolescents, and similar between sexes [2, 3]. Within 10 years of a first-time dislocation, 22.7% of people redislocate their patella [3]. The highest 10-year redislocation rate is 36.8% and occurs in 10 to 17 year-old girls [3]. When the patella dislocates laterally, the medial patellofemoral ligament is normally torn [4], making the knee painful and swollen, and patients usually attend hospital. Once the acute injury is managed, patient referral for exercise-based rehabilitation is recommended [5]. Surgery is mainly reserved for concurrent injuries and when non-surgical treatment is unsuccessful [5, 6]. Currently, recovery after non-surgical treatment is often incomplete with recurrent patellar dislocation and instability, reduced knee function, and later surgery commonly reported [7].

Although exercise-based rehabilitation after acute patellar dislocation is routine, limited high-quality evidence exists to guide rehabilitation practice [8]. In the absence of evidence, rehabilitation incorporating gluteal and thigh muscle strengthening, neuromuscular training to optimise lower limb alignment, and sports-specific training (where relevant), has been recommended [5, 9]. Our preliminary study showed a progressively challenging rehabilitation programme incorporating these features and strategies to support participant adherence to prescribed exercise was deliverable and associated with high participant-reported acceptability [10]. This type of rehabilitation programme could improve patient outcomes but has not been evaluated in a randomised controlled trial (RCT). Previous RCTs for people with other musculoskeletal conditions have also shown that multiple sessions of individually tailored exercise and advice were not more clinically effective than one session of advice, exercise, and provision of self-management materials [11, 12].

A full-scale RCT would determine which of these rehabilitation approaches is most clinically and cost-effective for people after acute patellar dislocation and provide high-quality evidence to guide rehabilitation provision for these patients treated in the UK National Health Service (NHS). Whether a full-scale trial is feasible is uncertain: the only previous RCT that compared exercise-based programmes for people after acute patellar dislocations had 52% loss to follow-up [13].

### Objectives

This study aims to determine the feasibility of conducting a full-scale RCT comparing supervised versus self-managed rehabilitation for people after acute patellar dislocation. Pilot objectives are to assess patients’ willingness to be randomised, the recruitment rate, adherence to the study intervention, retention, and to understand participants’ experience of recovery and the acceptability of the study interventions and follow-up methods.

## Methods

This protocol is reported following relevant sections of the Standard Protocol Items: Recommendations for Intervention Trials (SPIRIT) guidance for protocols of clinical trials (checklist available in Additional file 1) [14].

### Study design

**Fig. 1:**
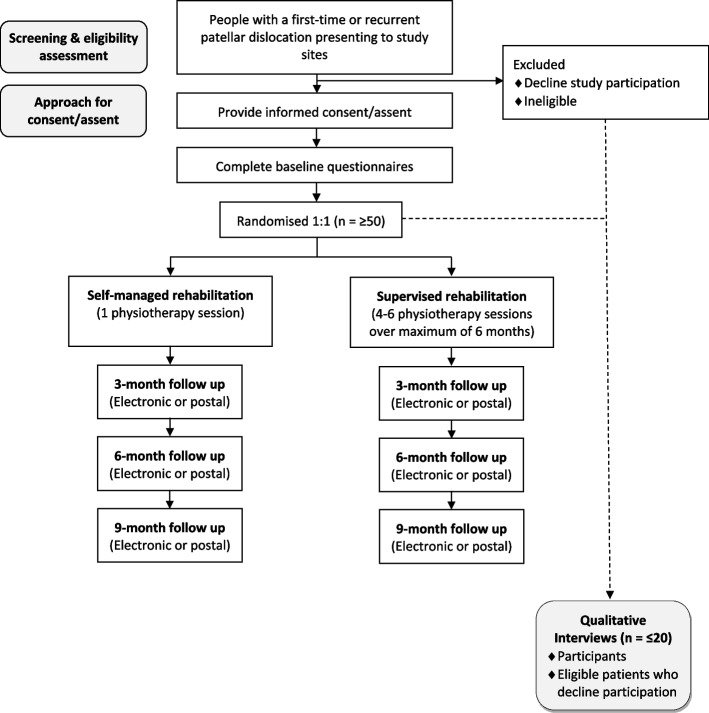
Participant flow through the study

Multicentre, two-arm, parallel-group, external pilot RCT with an embedded qualitative study comparing supervised versus self-managed rehabilitation for people aged ≥ 14 years with an acute first-time or recurrent patellar dislocation. Allocation to the study interventions will be 1:1. Figure 1 shows participant flow through the study.

### Setting

We will recruit participants from at least three English NHS hospitals, chosen considering their previous collaborations with our group, geographical location, and the sociodemographic characteristics of local populations.

### Eligibility criteria

Participants must be:

- Aged ≥ 14 years
- Have a first-time or recurrent patellar dislocation confirmed if the:
  - ○ Patellar dislocation was reduced by a healthcare professional or
  - ○ The patient reports a visible lateral patellar dislocation or sensation of the patella “popping out” of joint followed by reduction and the assessing clinician diagnoses a lateral patellar dislocation
- Participants aged ≥ 16 years must be willing and able to provide informed consent or for those aged < 16 years the parent/guardian must be willing and able to provide informed consent for the patient’s participation and the patient must be able to provide assent should they wish to do so

Exclusion criteria are:

-  > 21 days from injury
- Previous patellar stabilisation surgery on the affected knee
- Requires acute surgical intervention
- Contraindication(s) to participation in the study interventions
- Patient is unable to adhere to the study procedures
- Previously randomised into the study

For the rest of this report, “parent” refers to a parent or someone with parental responsibility for a patient/participant aged < 16 years.

### Recruitment

Potentially eligible patients will be identified in emergency departments, minor injuries units, and fracture/knee clinics. Appropriately qualified healthcare professionals will assess eligibility. Eligible patients (and parents for patients aged < 16 years) will be invited to discuss the study with a local researcher which may take place face-to-face or remotely by video/phone. The researcher will explain the study, provide a patient information sheet (an animated explainer video will also be available), and answer any questions. An age-appropriate patient information sheet will be available for patients aged < 16 years.

### Consent/assent

If agreeable, informed consent will be obtained face-to-face or by video/phone. For patients aged < 16 years, parental consent is required for participation. Patients aged < 16 years will be invited to provide assent, but a completed assent form is not necessary for participation. However, if the young person indicates on the assent form they do not want to participate they will not be included, even if their parent provides consent. Participants aged 15 years who turn 16 within the 9-month follow-up period will be contacted to have an informed consent discussion. If agreeable, informed consent will be obtained as described previously.

### Allocation

After providing informed consent/assent and completing baseline questionnaires, participants will be randomly allocated 1:1 to “supervised rehabilitation” or “self-managed rehabilitation” by researchers at study sites using an encrypted web-based service provided by Oxford Clinical Trials Research Unit. The randomisation sequence will be computer-generated and stratified by the study site and first-time/recurrent patellar dislocation (affected knee) with permuted blocks of varying lengths.

### Blinding

The nature of the interventions means blinding researchers, participants, or intervention providers to treatment allocation will not be possible. The trial statistician will also be unblinded. This should have limited impact on outcome data collection because most outcomes are participant reported and collected directly from participants.

### Interventions

We will describe the interventions fully in a separate report, so only a summary is included here.

Initial injury management will follow clinicians’ routine practice, but the knee splint and weight-bearing instructions (if any) provided at baseline will be recorded. Upon randomisation, researchers/clinicians will introduce a workbook (online version also available) to participants containing initial self-management advice and exercises targeting knee flexibility, balance, and leg strength, so participants can start their recovery immediately. Initial physiotherapy sessions will be up to 60 min and face-to-face (video sessions allowed if essential) within 3 weeks of randomisation. Registered NHS physiotherapists will provide the interventions. Participant-reported adherence to prescribed exercise will be recorded by follow-up questionnaire 3, 6, and 9 months after randomisation.

#### Self-managed rehabilitation

A single, one-to-one, physiotherapy session. After their normal clinical assessment, physiotherapists will re-emphasise relevant advice in the workbook to participants and provide any other advice deemed necessary. Physiotherapists will then prescribe one starting exercise in four different categories (knee bending, knee straightening, balance, and leg strengthening), each containing several exercises of progressive difficulty. Physiotherapists will emphasise the importance of exercise adherence and progression for recovery and, where relevant, safe return to sport. Physiotherapists will also use strategies to facilitate participant exercise adherence: participants will practice exercises and receive feedback, goal setting, action planning, and provide an exercise diary. Participants will then be discharged to continue their recovery independently using guidance in the workbook and online exercise videos. Participants who struggle with their exercises can initiate one follow-up phone/video/face-to-face physiotherapy session.

#### Supervised rehabilitation

Four to six, one-to-one, physiotherapy sessions over a maximum of 6 months. Follow-up sessions (face-to-face or video; phone only if essential) will be up to 30 min. The key difference in this intervention is the follow-up sessions which will enable physiotherapists to re-assess (including balance and objective leg strength assessment) participants and tailor advice and prescribed exercises as needed. Physiotherapists can prescribe up to five exercises per session from a pre-specified menu comprised of flexibility, leg strengthening, balance, and running exercises. Physiotherapists have more exercises to choose from in this intervention and can prescribe one “bespoke” exercise per session not on the exercise menu if deemed important to help participants reach their activity goal(s). One leg strengthening exercise must be prescribed per session following exercise prescription guidelines [15] because knee extensor strength deficits in the affected leg are common in this patient population [16]. Physiotherapists will use the same strategies to facilitate exercise adherence from “self-managed rehabilitation” with follow-up sessions also enabling physiotherapists to review participants’ goals and help participants problem solve if exercise adherence is problematic. At discharge, physiotherapists will advise participants about the importance of maintaining a healthy weight, leg strength, balance, and knee flexibility for long-term knee health.

#### Other healthcare treatment

Other healthcare treatments will continue as normal. Additional treatment participants receive for their patellar dislocation, including out-of-study physiotherapy and surgery, will be recorded through site reporting and participant follow-up questionnaires. Participants’ general practitioners (GPs) will be informed of their study participation because GPs can refer for physiotherapy.

### Outcomes

#### Pilot outcomes

To assess the:

- Willingness to be randomised: proportion of eligible patients approached who are randomised.
- Recruitment rate: number of participants recruited per month per site.
- Intervention adherence: proportion of participants allocated to “supervised rehabilitation” and “self-managed rehabilitation” attending at least four physiotherapy sessions and one physiotherapy session, respectively.
- Retention: proportion of participants that return 9-month Knee Injury and Osteoarthritis Outcome Score (KOOS_4_) outcome data (planned full-scale RCT primary outcome).

Progression criteria for quantitative pilot outcomes are presented in Table 1. A traffic light system [17] for each outcome will inform decision-making about the feasibility of a full-scale RCT. The trial management group will decide if a full-scale RCT is feasible based on quantitative progression criteria, whether any problems are considered resolvable, and qualitative study findings.

**Table 1:**
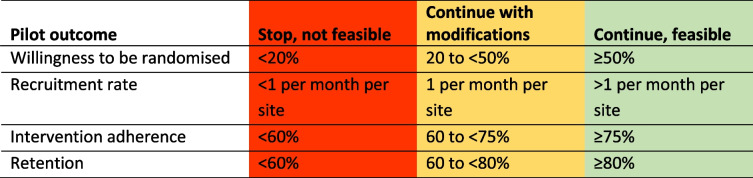
Progression criteria for quantitatively measured pilot outcomes

#### Exploratory clinical outcomes

To assess if the planned clinical outcomes for the full-scale RCT can be collected, we will collect them in this study. These include:

- Knee symptoms and function: assessed by the KOOS_4_ [18], a knee-specific 42-item participant-reported questionnaire widely used as the primary outcome in RCTs of people with knee injuries including patellar dislocations [19–21]. The KOOS_4_ is the average of four of the five domains (pain, other symptoms, function in sports and recreational activities, and knee-related quality of life). The additional domain is function in activities of daily living. Individual domains are scored 0 to 100 (higher scores better) and will be reported separately. Assessed at baseline (only “current” and not “pre-injury” scores), and 3, 6, and 9 months after randomisation. Because the KOOS asks participants about their knee during/in the “last week” and some participants may be randomised within one week of their injury, we will specify in the baseline questionnaire that if participants’ injury occurred “less than 1 week ago”, they should answer questions based on how their injured knee has been “since your injury”. This aims to ensure no pre-injury scores are included at baseline.
- Health-related quality of life: assessed by the EuroQol 5 Dimensions (EQ-5D-5L) [22], a generic participant-reported quality of life questionnaire comprised of five domains: mobility, self-care, usual activities, pain/discomfort, and anxiety/depression. Domains contain five response levels: “no problems, slight problems, moderate problems, severe problems, unable to/extreme problems”. Individual domain responses will be combined to create one overall utility score, ranging from -0.594 (worse than dead) to 1 (full health) for UK populations, by mapping data onto the EQ-5D-3L value set as recommended by the National Institute for Health and Care Excellence (NICE) [23]. If the new EQ-5D-5L valuation is completed and recommended for use by NICE before data analysis, we will calculate EQ-5D-5L utility scores using both approaches. Participants also rate overall health on a visual analogue scale anchored at 0 (“worst health you can imagine”) and 100 (“best health you can imagine”). Assessed at baseline (only “current” scores) and 3, 6, and 9 months after randomisation.
- Return to main pre-injury sport/physical activity: participant-reported percentage return to their main pre-injury sport or physical activity using a visual analogue scale anchored at 0% (“not participating at all in your main sport or physical activity”) and 100% (“You have fully returned to your main sport or physical activity”). Assessed at baseline, and 3, 6, and 9 months after randomisation.
- Global rating of change: participant-reported change in their affected knee compared with when they entered the study, measured on a seven-point Likert scale (a lot worse, moderately worse, a little worse, no change, a little better, moderately better, a lot better). Assessed 3, 6, and 9 months after randomisation.
- Complications: any serious adverse events (SAEs) that occur will be processed following the clinical trials unit’s standard operating procedures. Foreseeable SAEs and complications not defined as serious will be collected from site reporting and participant follow-up questionnaires 3, 6, and 9 months after randomisation. Expected complications include:
  - ○ Deep vein thrombosis or pulmonary embolism
  - ○ Surgery to the injured knee (not meeting SAE criteria)
  - ○ New ipsilateral or contralateral patellar dislocation (not meeting SAE criteria)
  - ○ Increased knee pain and/or swelling during or after completing the intervention exercises that requires a healthcare professional consultation
  - ○ Any new or exacerbated medical condition that started during or after completing the intervention exercises that requires a healthcare professional consultation

New patellar dislocations are defined as those reported by participants that require hospital or GP attendance, or if there is a documented patellar dislocation diagnosis in a participant’s medical records that occurred after the index injury. Site investigators will be asked to check participants’ medical records if any unreported patellar dislocations or related knee surgery occurred during the 9-month follow-up period. Safety reporting will start once a participant is randomised and end 9 months after randomisation.

We will also investigate the development of health resource use case reports forms for the full-scale trial.

Table 2 shows participants’ schedule of events.

**Table 2:**
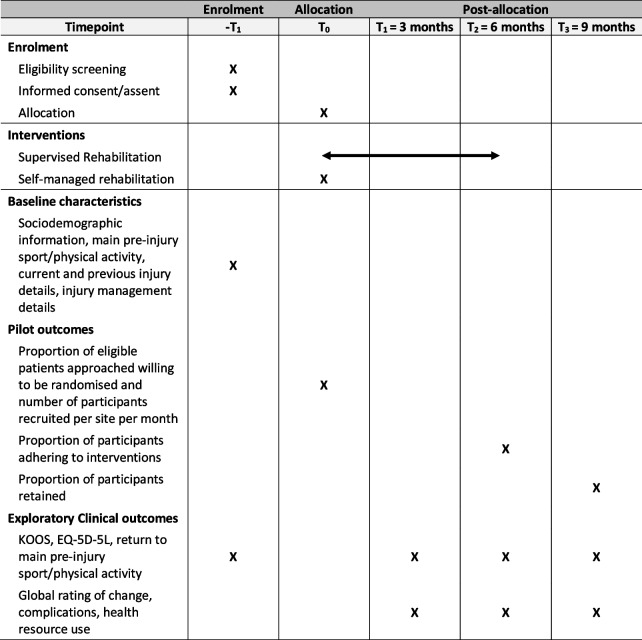
Schedule of events for participants in the pilot RCT

### Sample size

Retention is the main uncertainty for the full-scale trial and was the main driver of the sample size calculation. We aim to recruit at least 50 participants which will enable us to estimate retention with a 95% confidence interval ± 11% using Wilson’s methods if it is 80% or higher [24].

### Data collection

#### Baseline data collection

Baseline data collection will be electronic and includes sociodemographic data, injury and previous injury details, main pre-injury sport/physical activity, and the knee splint and weight-bearing instructions provided. Participants will also complete the KOOS, EQ-5D-5L and return to main pre-injury sport/physical activity questionnaire.

#### Physiotherapist treatment and training logs

We will record physiotherapists’ experience and intervention session details, i.e. if the participant attended, session number, session duration, delivery of core intervention components, prescribed exercises, additional treatment(s) provided, and if the participant was discharged.

#### Three, 6, and 9-month follow up

Participants aged ≥ 16 years: 3, 6, and 9 months after randomisation, participants will be invited to complete electronic questionnaires by email and/or text, according to their preference. Postal questionnaires will be available if required. Participants who do not complete questionnaires will be sent automated reminders. If required, participants will be contacted directly by email, text, or telephone to encourage follow-up, obtain missing data, and resolve data queries.

Participants aged < 16 years: Questionnaires and automated reminders will be sent to parents. These will also be sent to participants if parental consent is obtained. All other contacts to encourage follow-up, obtain missing data, or resolve data queries will be through parents. Participant assent is not required to receive questionnaires and automated reminders, but if the participant indicates on the assent form they do not want to receive questionnaires and automated reminders, these will be sent to the parent only.

#### Withdrawals

Participants and parents (for participants aged < 16 years) will be informed participation is voluntary and they can withdraw at any time without explaining why and without affecting the quality of their clinical care. Participants will be able to withdraw from treatment but participate in data collection. They will not be able to withdraw data obtained before their withdrawal as this data is required for the intention-to-treat analysis and analysis of safety. Types of withdrawal and withdrawal reasons (if provided) will be recorded.

### Statistical analysis

We will summarise quantitative data using descriptive statistics and appropriate summary statistics e.g., means and standard deviations and/or medians and interquartile ranges. We will report categorical data using counts and percentages. Recruitment rate will be summarised as mean recruitment per site month and 95% exact Poisson confidence interval will be generated. Other quantitative pilot outcomes (including retention) will be expressed as proportions with 95% confidence intervals at the timepoints they are assessed. Confidence intervals for proportions will be calculated using Wilson’s method [25]. As this is a pilot trial, no inferences about the comparative effectiveness of the study interventions will be made. Randomised participant data will be grouped according to their treatment group allocation, regardless of the treatment they receive. We will seek to minimise missing data through careful data management. No imputation of missing data is planned.

### Qualitative study

This aims to understand the acceptability of the interventions and follow-up methods to participants which are key uncertainties of the full-scale trial. This qualitative study will also enhance our understanding of participants’ experience of injury and recovery after acute patellar dislocation.

#### Data collection

We will conduct one, face-to-face (or by video/telephone if required), semi-structured interview with up to 20 participants at a mutually agreeable location. We will purposively sample participants for variation in treatment allocation, age (aged ≥ 16 years versus aged < 16 years) and completed/lost to follow-up to obtain a breadth of experience. Eligible patients who decline study participation will also be interviewed to explore their experience of injury and recovery. A sensitising topic guide will be developed with patient and public involvement (PPI) partners drawing on their experience and current evidence, and refined iteratively. We will use open-ended questions to elicit participants’ experience.

During the consenting process for the pilot RCT, participants or parents (for participants aged < 16 years) will indicate if we can contact them about interview participation. Eligible patients or parents (for eligible patients aged < 16 years) who decline to participate in the pilot RCT will also be asked for permission to contact them about interviews. Where permitted, we will contact participants or parents (for those aged < 16 years) to explain the purpose of interviews, provide a patient information sheet, and answer any questions. A young person patient information sheet will be available for patients aged < 16 years. Informed consent/assent will be obtained face-to-face or by video/phone as described for the pilot RCT.

#### Data analysis

We will conduct and analyse interviews concurrently. Interviews will be audio-recorded and transcribed verbatim. We will use NVivo software to help organise the data. Field notes will be taken after interviews to record additional contextual information and the interviewer’s reflections. Data will be analysed iteratively using thematic analysis [26]. We will keep an audit trail of decisions made during analysis and support findings with de-identified direct participant quotations.

### Quality assurance/study monitoring

There is no trial steering, data, or safety monitoring committees because this is a low-risk pilot study. The chief investigator (CF) will be responsible for day-to-day study management, with support from a senior trial manager, because he is completing this study as part of a DPhil (PhD). A trial management group will meet monthly to oversee study set-up, conduct, and any safety issues. Quality control procedures will be undertaken during recruitment and data collection to ensure the research is conducted, generated, recorded, and reported in compliance with the protocol, good clinical practice (GCP), and ethics committee recommendations. The chief investigator and trial manager will develop data management and monitoring plans. We will monitor intervention delivery through periodic site visits and/or audio recording treatment sessions, and by reviewing physiotherapist-completed treatment logs. Sites will receive feedback from quality assurance checks and additional training will be provided, if required.

### Data management and access

Data management and access will follow the clinical trial unit’s standard operating procedures and GCP guidelines. Study data will be collected using REDCap (Research Electronic Data Capture). Data collected on paper forms or during phone calls will be entered directly into the study database. Qualitative interviews will be digitally audio-recorded on encrypted devices, electronically transcribed, and pseudonymised. Data will be stored on secure servers at the University of Oxford and accessible to authorised personnel only.

### Ethics and dissemination

This study was prospectively registered on the ISRCTN registry (ISRCTN14235231) on 09 August 2022. The East of Scotland Research Ethics Service provided ethical approval (Research Ethics Committee reference: 22/ES/0035).

### Protocol amendments

Substantial amendments will be submitted for approval from the Research Ethics Committee and Health Research Authority. Any substantial amendments to this protocol will be described in the study report.

### Patient and public involvement

The National Institute for Health and Care Research (NIHR) GenerationR Liverpool Young Persons Advisory Group (funded by the NIHR Alder Hey Clinical Research Facility), and two adults with previous patellar dislocations helped design this study. “Self-managed rehabilitation” allows one optional follow-up session if participants are struggling with prescribed exercise following advice that this would improve intervention acceptability. Initial physiotherapy sessions are face-to-face based on feedback that this would help participants perform initial exercises correctly, whereas follow-up sessions can be remote. “Supervised rehabilitation” was originally called “best-practice feedback” but was renamed following comments that the original name inferred this intervention was superior. Patient reported outcome measures were chosen following unanimous feedback that pain or restoring pre-injury activity levels/function were the most important outcomes. We will use electronic follow-up and exercise videos following advice that these would improve retention and exercise adherence, respectively. Adult patient information sheets were reviewed by adult PPI members. Young person patient information sheets, assent forms, and the explainer video transcript were reviewed by GenerationR members.

### Dissemination policy

The pilot RCT and embedded qualitative study will be reported following the Consolidated Standards of Reporting Trials (CONSORT) extension to randomised pilot and feasibility trials guidelines [27] and submitted for publication in an open-access peer-reviewed journal regardless of the study results. A standalone qualitative study report will be submitted for publication in a peer-reviewed journal if data are sufficient. We will determine authorship using the International Committee of Medical Journal Editors guidelines [28]. Other contributors will be acknowledged. Patient information sheets will inform participants that a summary of the study results will be available on the study website.

## Discussion

Non-surgical exercise-based rehabilitation is routinely provided for people with an acute patellar dislocation, but no high-quality evidence exists to guide rehabilitation practice and treatment outcomes vary. A full-scale trial comparing different rehabilitation approaches would provide high-quality evidence to guide rehabilitation provision for people with a patellar dislocation treated in the NHS. This study will address key uncertainties over the feasibility of conducting this future full-scale trial. This follows the current Medical Research Council and NIHR framework for developing and evaluating complex interventions [29]. Study results are expected to be available in Autumn 2024.

## Supplementary Information

**Additional file 1.** SPIRIT 2013 Checklist: Recommended items to address in a clinical trial protocol and related documents.

## Data Availability

Not applicable.

## Abbreviations

CONSORT: Consolidated Standards of Reporting Trials
EQ-5D-3L: EuroQol 5 Dimensions 3 Levels
EQ-5D-5L: EuroQol 5 Dimensions 5 Levels
GCP: Good Clinical Practice
GP: General practitioner
KOOS: Knee Injury and Osteoarthritis Outcome Score
NHS: National Health Service
NICE: National Institute for Health and Care Excellence
NIHR: National Institute for Health and Care Research
PPI: Patient and public involvement
RCT: Randomised controlled trial
REDCap: Research Electronic Data Capture
SAE: Serious adverse event
SPIRIT: Standard Protocol Items: Recommendations for Intervention Trials
